# TRACE: An Unbiased Method to Permanently Tag Transiently Activated Inputs

**DOI:** 10.3389/fncel.2020.00114

**Published:** 2020-05-12

**Authors:** Nathalie Krauth, Valentina Khalil, Meet Jariwala, Noemie Mermet-Joret, Anne-Katrine Vestergaard, Marco Capogna, Sadegh Nabavi

**Affiliations:** ^1^Department of Molecular Biology and Genetics, Aarhus University, Aarhus, Denmark; ^2^DANDRITE, The Danish Research Institute of Translational Neuroscience, Aarhus, Denmark; ^3^Department of Biomedicine, Aarhus University, Aarhus, Denmark; ^4^Center for Proteins in Memory—PROMEMO, Danish National Research Foundation, Aarhus, Denmark

**Keywords:** neural circuits, optogenetics, viral tracing, innate fear, mouse behavior

## Abstract

A fundamental interest in circuit analysis is to parse out the synaptic inputs underlying a behavioral experience. Toward this aim, we have devised an unbiased strategy that specifically labels the afferent inputs that are activated by a defined stimulus in an activity-dependent manner. We validated this strategy in four brain circuits receiving known sensory inputs. This strategy, as demonstrated here, accurately identifies these inputs.

## Introduction

Most brain regions receive a large number of neuronal inputs; this poses a challenge to circuit analysis since only a fraction of these inputs convey information for a defined behavior. Identifying these inputs is crucial to gaining an insight into the neural circuits that underlie the behavior. Currently, there is no direct way of achieving this goal. A common strategy is to use retrograde tracing viruses which are designed to identify all the inputs to the region of interest (Wickersham et al., 2007; Tervo et al., 2016; Luo et al., 2018). To identify the specific inputs, the researcher must then rely on a combination of trial and error, an educated guess, and previous findings. A more efficient way would be to label only the inputs that are activated by the stimuli. Recent developments in the use of immediate-early gene promoters offer such an opportunity (Guenthner et al., 2013; DeNardo and Luo, 2017). The underlying mechanism is simple: a gene of interest is expressed under the control of an activity-dependent promoter such as Arc or cfos. The neurons that are activated by the behavioral experience will express the gene of interest such as a fluorescent marker. This approach, as currently used, however, does not reveal the active inputs.

Here, we introduce a novel approach, Tracing Retrogradely the Activated Cell Ensemble (TRACE), which selectively labels the afferent inputs that are activated by a defined stimulus and project to the region of interest. It combines activity-dependent labeling with virus-mediated retrograde tracing.

This approach is unbiased, as it does not rely on pre-existing knowledge of candidate regions and offers high temporal (within 6 h; Ye et al., 2016; DeNardo et al., 2019) and spatial (cellular scale) resolution.

## Methods

### Animals

ArcCreERT2 heterozygous mice B6.Cg-Tg(ArcCre/ERT2)MRhn/CdnyJ (JAX stock #022357) from Jackson labs were used in the optical activation experiments and the odor-driven innate fear experiments. In the foot-shock experiments cfosCreERT2 heterozygous mice B6.129(Cg)-Fostm1.1(Cre/ERT2)Luo/J (JAX stock #021882) from Jackson labs were used.

All mice were given food and water *ad libitum* and were kept at a 12 h light/dark cycle, with the light being on during the daytime. All behavioral tests were performed during the day-time, however, the animals were kept in isolation in a dark room 24 h before and 72 h after the testing days. Odor driven innate fear experiments were only performed with male mice since it was observed that the female mice did not react to 2,3,5-trimethyl-3-thiazoline (TMT) in the same manner as their male littermates (data not shown). Both the optogenetic activation and shock-induced circuit experiments were performed with mice of both sexes and mixed test groups, as no difference was observed. All procedures involving animals were approved by the Danish Animal Experiment Inspectorate.

### Viral Vectors and Tracers

The AAV were obtained from the viral vector core facility at the University of Zurich in Switzerland. Virus titers were the following: 4.5 × 10^12^ for AAV-hSyn1-dlox-EGFP(rev)-dlox-WPRE-hGHp(A; serotype AAV2-retro); 5.0 × 10^12^ for AAV-hSyn-oChIEF-tdTomato (Serotype AAV8); 1.0 × 10^13^ for AAV-hSyn-oChIEF-td tomato (serotype AAV2-retro); 4.8 × 10^12^ for AAV-hSyn1-dlox-mCherry(rev)-dlox-WPRE-hGHp(A; serotype AAV2-retro); 7.0 × 10^12^ for AAV-hSyn1-dlox-mCherry(rev)-dlox-WPRE-hGHp(A; serotype AAV9).

### Stereotactic Surgery

Mouse surgeries were performed on 5-week-old mice to ensure low background labeling. The mice were anesthetized using a mix of 0.05 mg/ml of Fentanyl [(Hameln, 007007) 0.05 mg/kg], plus 5 mg/ml of Midazolam [(Hameln, 002124) 5 mg/kg] and 1 mg/ml of Medetomidine [(VM Pharma, 087896) 0.5 mg/kg; FMM] and surgeries were performed using a stereotaxic frame (Kopf Instruments). The scalp was opened using scissors and holes were drilled using a Foredom high-speed drill (Foredom Electric Co, K.1070-22). Coordinates were normalized to a bregma-lambda distance of 4.21 mm. In the case of viral injections, the animals were injected with a pulled 1 mm glass pipette using a Picospritzer or Nanoject III (Drummond) containing the respective virus mix for each experiment. Five hundred nanoliters μl of virus mixtures were injected per location unless otherwise stated in the main text.

Viral injection coordinates for the optogenetic stimulation experiments were: lateral amygdala (LA) [from bregma: anterior-posterior (AP), −1.6 mm; medio-lateral (ML), 3.45 mm; and dorsal-ventral (DV) from the skull, −4.0 mm and TeA/Ect (AP, −2.5 mm and −3.0 mm; ML, 4.3 mm; DV, −2.0 and −2.4 mm)]. The fiber implant [0.22 numerical aperture (NA), 200 μm core diameter] was placed above the LA (AP, −1.5 mm; ML, 3.35 mm; and DV, −3.9 mm). Viral injection coordinates for the odor-activated innate fear experiments were: cortical amygdala [(CoA) (AP, −1.6 mm and −1.7 mm; ML, 2.7 mm; DV, −5.8 mm)]. Viral injection coordinates for the shock activated experiments were: lateral habenula [(LHb) (AP, −1.45 mm; ML, 0.3 mm; DV, −2.8 mm)] and nucleus accumbens [(NAcc) (AP, +1.5 mm; ML, 1.0 mm; DV, −4.5 mm)].

After the surgery, the wound was sutured and the mice received 200 μl of a local anesthetic (lidocaine 2%) to minimize pain from the surgery. The mice were given an antidote mix of 0.4 mg/ml Naloxone [(B. Braun, 115241) 1.2 mg/kg], plus 5 mg/ml of Atipamelozone Hydrochloride (2.5 mg/kg) and 0.5 mg/ml of Flumazenil [(Hameln, 036259) 0.5 mg/kg] to reverse the anesthesia and the animals were allowed to recover on a heating pad. Buprenorphine [Temgesic (Indivior UK Limited, 521634) 0.3 mg/ml] was added to the drinking water as an analgesic in the 2 days following surgery. The mice were allowed to recover for 2 weeks before behavioral tasks. The animals were checked daily after surgery.

All the viral injection sites and the optic fiber implants were confirmed histologically and the animals were excluded when the injection sites or the optic fiber implantation were misplaced.

### Behavioral Testing

#### Optogenetic Stimulation of LA

ArcCreERT2 mice were habituated to the plugging of the laser patch cord for 5 days while exploring the testing cage for 10 mins. Following this training, the mice were intraperitoneally (i.p.) injected with saline daily to habituate them for tamoxifen injections. One day before the testing day, the mice were moved to the dark housing, where they were individually housed until 72 h after the testing. Optogenetic stimulation was performed in the home cages using a laser with a 450 nm wavelength (Doric lenses) and a dual fiber optic patch cord (Doric Lenses, 0.22 NA, 200 μm core diameter). To induce antidromic spikes, we used a high-powered laser stimulation protocol (Adhikari et al., 2015) consisting of 10 trains of light (each train having 100 pulses of light, 5 ms each, 100 Hz at 50–80 mW) at 90-s inter-train intervals controlled with a pulse generator (Pulse Pal, Open Ephys). IP injection of 4-hydroxytamoxifen (4-OHT; 10 mg/kg) was performed 2 h after the optical stimulation. 4-OHT was prepared following Ye et al. (2016).

#### Odor Driven Innate Fear

ArcCreERT2 mice were habituated to the context consisting of an open field arena (45 cm × 35 cm) containing a small dark chamber (16.5 cm × 10 cm) in the center. The dark chamber had a single opening on the side (Genné-Bacon et al., 2016). Seven sessions of habituation were performed in 3.5 days, twice a day, and each session lasted 10 min (performed during daylight). At the beginning of each session, the mice were placed randomly around the dark chamber. Between the morning session and the afternoon session, the mice were habituated to intraperitoneal injections. One day before the testing, the mice were moved to the dark housing room, where they were kept individually housed until 72 h after the testing. For both phosphate buffer saline (PBS) and TMT (Sigma), 20 μl of the solution was pipetted onto a filter paper placed in the middle of the dark chamber. The TMT had previously been diluted 1:1 with PBS to lower the strength. The test group was exposed to PBS in the dark chamber in the morning and to TMT in the afternoon. The control group was exposed to PBS during both test sessions. The mice were positioned in front of the entrance of the dark chamber for the testing sessions. IP injection of 4-OHT (10 mg/kg) was performed 2 h after TMT exposure. The behavior was recorded and analyzed manually with a video tracking system (ANY-maze).

#### Shock-Activated Circuit

cfosCreERT2 mice were habituated to the context with five daily sessions of habituation. Habituation was performed by allowing the mice to explore the testing chamber for 10 min every day. The mice were further habituated to i.p injections for 10 mins every day. One day before the testing day, the mice were moved to dark animal housing, where they were kept until 72 h after the testing. On the testing day, the mice in the test group were placed in an ANY-maze controlled Fear Conditioning System (Ugo Basile). Mice received 20 random shocks (0.5 mA, 2 s long) distributed in 10 min of testing. The i.p. injection of 4-OHT (10 mg/kg) was performed 2 h after testing. The control group remained in the home cage but were also habituated to i.p. injections and were injected with 4-OHT on the testing day.

### Perfusion and Immunohistochemistry

The animals were anesthetized with FMM and euthanized by transcardial perfusion with 50 ml of PBS (with 50 mg/ml heparin), followed by perfusion with 50 ml of freshly prepared 4% Paraformaldehyde (PFA). The brains were extracted from the skull and stored in PFA for 2 days at +4°C. Brains were sliced into 80 μm thick slices in PBS on a Leica vibratome.

To enhance the fluorescent signals, an immunohistochemical staining was performed for the EGFP labeling in the odor driven innate fear and shock activated experiments. Antibody used was an EGFP rabbit [Invitrogen (CAB4211)] primary antibody with 1:1000 dilution and 72-h incubation. Alexa Fluor 488 goat anti-rabbit [Invitrogen (A-11008)] was used as the secondary antibody with 1:1000 dilution with 24-h incubation. Nuclear staining was done using 1:1000 dilution of DAPI for 30 min. Brain slices were mounted on glass slides with coverslips using Fluoromount G (Southern Biotech).

### Imaging and Cell Count

**Figure 1:**
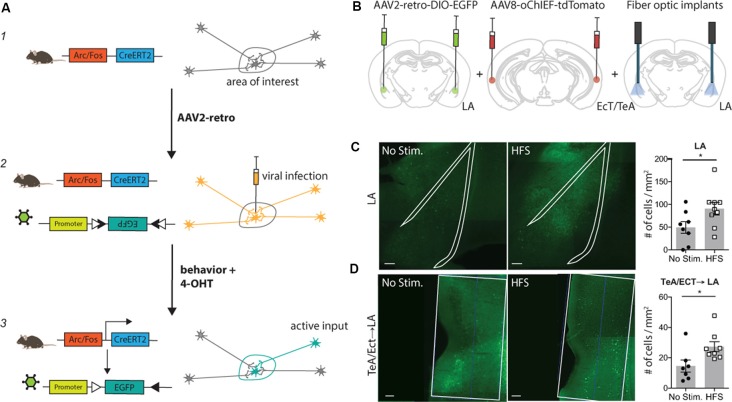
Tracing Retrogradely the Activated Cell Ensemble (TRACE) method. TRACE labeling of afferent cortical inputs to lateral amygdala (LA) after high-frequency stimulation (HFS). **(A)** Schematic of the TRACE method: (1) a transgenic mouse expresses the tamoxifen-inducible CreERT2-recombinase under the Arc or cfos promoter. (2) The AAV2-retro infects the cells in the area of interest and is also taken up by cellular projections of other areas to this region. The AAV2-retro has a floxed EGFP, which will not be expressed in this state. (3) Upon 4-hydroxytamoxifen (4-OHT) injection, the CreERT2 recombination occurs in active cells. Consequently, EGFP is expressed in all active cells that were previously infected with the virus. This causes labeling of active cells in the area of interest as well as in their respective inputs. **(B)** Experimental schematic. AAV2-retro-DIO-EGFP was injected into the LA, while the TeA (temporal association area)/Ect (ectorhinal cortex) were injected with the channelrhodopsin carrying virus AAV8-oChIEF-tdTomato. The optic fibers were placed above the LA to activate the channelrhodopsin infected projections from TeA/Ect to the LA. 4-OHT was injected 2 h after HFS. The quantification of active labeling in the LA and TeA/Ect was performed 1 week after the HFS treatment. **(C)** Exemplary images of the LA, outlined with the white line, in mice receiving HFS or no stimulation. Green shows activity-mediated EGFP fluorescent expression. Graph (right panel) shows the quantification of the fluorescently-tagged neurons. Labeling in the LA is significantly higher in the optically-activated animals [unstimulated group *n* = 8, mean ± SEM, HFS group *n* = 9; *p*-value = 0.0474 (unpaired *t*-test); scale bar: 100 μm]. **(D)** Representative images of the TeA/Ect, outlined with the white line, in mice receiving HFS or no stimulation. Green shows activity-mediated EGFP fluorescent expression in the LA-projecting neurons within TeA/Ect. The graph shows the quantification of the fluorescently-tagged neurons. Activity-dependent retrograde labeling is significantly higher in the activated projections from TeA/Ect to LA [unstimulated group *n* = 7, HFS group *n* = 8; *p*-value 0.014 (Mann–Whitney test); scale bar: 100 μm]. *p < 0.05.

**Figure 2:**
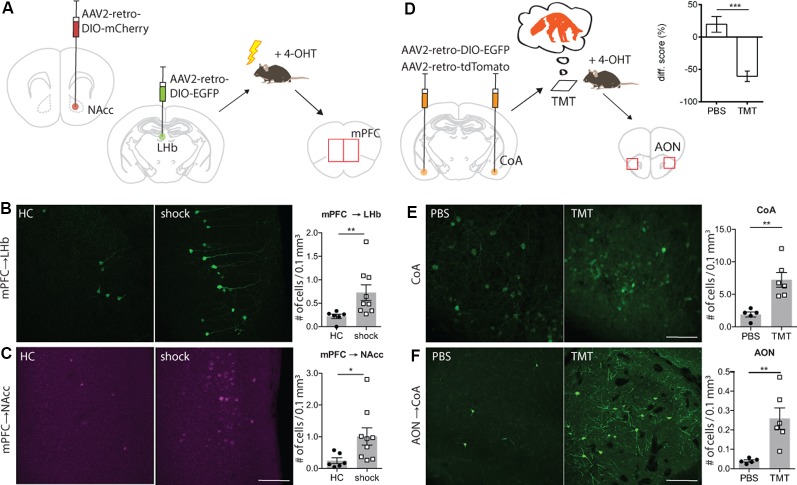
TRACE identifies inputs activated by aversive experiences. **(A)** Experimental schematic. Injection of AAV2-retro-DIO-EGFP into the left hemisphere lateral habenula (LHb). Injection of AAV2-retro-DIO-mCherry into the right hemisphere NAcc. Two weeks after virus infection of LHb and nucleus accumbens (NAcc), a group of animals was exposed to 20 mild electric foot-shocks within a 10-min session while a control group remained in their home cages. 4-OHT was injected 2 h after testing. The evaluation of active labeling in LHb, NAcc, and medial prefrontal cortex (mPFC) in both hemispheres was performed 1 week after testing. **(B)** Magnified images of the mPFC in mice receiving foot-shock. Green shows activity-mediated EGFP fluorescent expression in the LHb-projecting neurons within mPFC. As graph shows (mean ± SEM), TRACE-mediated labeling in the LHb-projecting neurons in the mPFC of animals exposed to the foot-shocks is significantly higher than the home cage group [home cage group *n* = 6, shock group *n* = 9; *p*-value = 0.0016 (Mann–Whitney test); scale bar: 100 μm]. **(C)** Same as **(B)**, for the NAcc-projecting neurons in the mPFC [home cage group *n* = 6, shock group *n* = 9; *p*-value = 0.012 (Mann–Whitney test)]. Color-converted magenta depicts the activity-mediated mCherry fluorescent expression. **(D)** Experimental schematic. Injection of AAV2-retro-DIO-EGFP and AAV2-retro-oChIEF-tdTomato into the cortical amygdala. Three weeks after virus infection of cortical amygdala (CoA), the animals were exposed to the 2,3,5-Trimethyl-3-thiazoline (TMT) odor, followed by 4-OHT injection 2 h later. Evaluation of active labeling in CoA and anterior olfactory nucleus (AON) was performed 2 weeks later. The graph shows the time spent within the chamber (% of total time) comparing habituation vs. test sessions for both phosphate buffer saline (PBS) and TMT groups [PBS group *n* = 7; TMT group *n* = 9; 0.0003 (Mann–Whitney test)]. **(E)** Magnified images of the CoA in mice exposed to PBS or TMT. As shown in the right graph, activity-dependent EGFP expression is significantly higher in TMT-exposed mice [PBS exposed *n* = 5, TMT exposed *n* = 6; *p*-value = 0.0043 (Mann–Whitney test); scale bars: 100 μm]. **(F)** Magnified images of the AON in mice exposed to PBS or TMT. TRACE-mediated labeling in the CoA-projecting neurons in the AON of animals exposed to TMT is significantly higher [PBS group *n* = 5, TMT group *n* = 6; *p*-value = 0.0043 (Mann–Whitney test)]. *p < 0.05, **p < 0.01.

Imaging from the brain slices of the optogenetic stimulation (Figure 1 and Supplementary Figure S1A) was done using the ZEN software (ZEISS) and a ZEISS Apotome microscope (Axio Imager.M2). Tile images were taken from the whole brain slice using a 10× objective (Apochromat 10×/0.45 M27). Filter excitation and emission wavelengths for Alexa 488 were 450–490 nm, and 500–550 nm. The parameters for DAPI were 335–383 nm 420–470 nm. Imaging for the odor driven innate fear and shock-activated tracing experiments (Figure 2, Supplementary Figures S1B,C, S3) was performed using the ZEN software (ZEN 2.5, blue edition) and a ZEISS confocal microscope (LSM 780/Axio Observer.Z1m). The images were taken as z-stack images (z-step size = 2 μm) in the respective areas using a 20× objective (Plan-Apochromat 20×/0.8 M27) or 10× objective (Apochromat 10×/0.45 M27) at the resolution of 1,024 pixels × 1,024 pixels. Alexa 488 was excited at 488 nm and detected through a bandpass filter of 509–605 nm, while the DAPI was stimulated at 405 nm and detected through a 426–488 nm bandpass filter. mCherry fluorescence was excited at 543 nm and detected through a 580–648 nm bandpass filter. The parameters for excitation and detection of tdTomato were 543 nm and 595–649 nm. Samples were imaged in a sequential acquisition mode to avoid bleed through in the other channels. To avoid photoconversion of mCherry fluorescence, the imaging of the DAPI was always performed after imaging of the tdTomato and Alexa 488 (Kremers et al., 2009; Guenthner et al., 2013). Cell counting for the optogenetic activation experiment was performed blindly and manually in ImageJ. Cell counting for odor driven innate fear and shock-activated circuit experiments were performed blindly and semi-manually using the cell count function in Imaris (Bitplane). The z-stacks were merged automatically and an automatic spot-detection function (“Best Quality,” defined by the pixel intensity median according to the user manual) was used. Before counting, the experimenter measured the radius of few cells randomly selected and the average was used as expected radius. After the automatic analysis was performed, the experimenter adjusted the lower and the higher threshold that was calculated by Imaris to remove false positive or false negative signals that were detected by the software.

### Statistics

Statistical analyses were performed in PRISM 6 (GraphPad). Data from male and female subjects were pooled in all experiments apart from the TMT experiment, which only included male subjects. Data were tested for normality with the D’Agostino-Pearson normality test, and a parametric test was used for the data that presented a normal distribution. If not, a non-parametric test (Mann–Whitney test) was performed. The statistical method and corresponding *p*-values are reported in the figure descriptions.

## Results

TRACE is based on two recently developed methods: (1) labeling of active neurons wherein neurons express the tamoxifen-inducible CreERT2-recombinase under the control of an activity-dependent promoter such as Arc or cfos (Guenthner et al., 2013; Kawashima et al., 2013; Denny et al., 2014); and (2) labeling with a retrograde virus, such as AAV2-retro, that expresses a marker gene in a recombination-dependent manner (Tervo et al., 2016). In this work, we chose AAV2-retro as it is one of the most efficient retrograde viruses available, also showing little toxicity (Tervo et al., 2016). Importantly, TRACE can be adapted to any DNA-based retrograde virus. TRACE works as follows: first, an AAV2-retro carrying a Cre-dependent marker is injected into a target brain area. Then, the virus is taken up by post-synaptic neurons in the target region as well as by the axons of the projecting neurons, but the neurons expressing the virus remain unlabeled in the absence of tamoxifen and neuronal activity. As the animals are exposed to a behavioral experience, only the projecting neurons activated in the short period of the behavior express CreERT2. We then inject 4-hydroxytamoxifen (4-OHT) to induce CreERT2 recombinase translocation into the nucleus and recombination of the marker genes. This results in a permanent expression of the marker genes in the active neurons projecting to the target area (Figure 1A). To demonstrate the input specificity of this method, we used this approach in four different behaviorally-relevant neuronal circuits.

## Trace Labeling of Afferent Cortical Inputs to Lateral Amygdala After High-Frequency Stimulation (Hfs)

First, we chose to apply our method to label afferent inputs from the temporal association area and ectorhinal cortex (TeA/Ect) to the LA, since this is a well-characterized pathway with clear-cut behavioral significance (Nabavi et al., 2014). We injected an AAV expressing a variant of the light-activated channel ChR2, oChIEF, into the TeA/Ect and an AAV2-retrovirus expressing EGFP in a Cre-dependent manner in the LA of ArcCreERT2 mice. A fiber optic was placed above the LA to deliver optical stimulation (Figure 1B and Supplementary Figure S1A). Three to four weeks after the viral injection, multisensory inputs to the LA were stimulated with high-frequency light pulses for the induction of antidromic action potentials. Following optical stimulation, mice were injected with 4-OHT. We observed significantly more EGFP-tagged neurons in the post-synaptic neurons in the LA (Figure 1C and Supplementary Figure S4A) and, more importantly, in presynaptic neurons in the TeA/Ect of the mice receiving optical activation compared to non-stimulated mice (Figure 1D and Supplementary Figure S4B).

## Trace Identifies Inputs Activated by Aversive Experience

Next, we explored whether TRACE could identify the inputs for an aversive foot-shock. We chose this particular stimulus since it has been widely used in studies on associative learning and valence experiences (Ye et al., 2016). For this, we focused on the pathway from the medial prefrontal cortex (mPFC) to the LHb and the Nacc, since these inputs are known to convey an aversive signal for a foot-shock to these targeted areas (Ye et al., 2016; Kim et al., 2017). We injected AAV2-retroviruses expressing two different fluorescent markers in a Cre-dependent manner in the LHb (AAV2-retro-DIO-EGFP) and the NAcc (AAV2-retro-DIO-mCherry) of cfosCreERT2 mice (Figure 2A and Supplementary Figure S1B). Two weeks after the virus injection, mice were divided into two groups. The test group received a series of foot-shocks followed by injection of 4-OHT, whereas the control group received 4-OHT in their home cages. Animals receiving foot-shocks had significantly higher fluorescent marker expression in the mPFC compared to the control group (Figures 2B,C), consistent with the previous reports on the aversive nature of the mPFC inputs to the LHb and NAcc (Ye et al., 2016; Kim et al., 2017).

## Trace Identifies Inputs Activated by Innate Aversive Experience

Finally, we chose to characterize inputs onto the cortical amygdala (CoA) driven by an innately aversive odor, which is likely to recruit long-range projections from the olfactory nuclei (Root et al., 2014). As a stimulus, we used TMT, a volatile component found in fox secretions, that is known to activate the inputs from the olfactory cortex to the CoA (Rosen et al., 2015). The odor-driven activation of these inputs to the CoA induces an innate avoidance behavior (Figure 2D and Supplementary Figure S2). We injected AAV2-retro expressing Cre-dependent EGFP in the CoA of ArcCreERT2 mice (Figure 2D). To verify the injection sites, additionally, we injected an AAV2-retro Cre-independent red fluorescent marker (Supplementary Figure S1C). Three weeks after the virus injection, mice were divided into two groups. The test group was exposed to TMT, and subsequently, they received a dose of 4-OHT. The control group received the same treatment as the test, except that TMT was replaced with PBS. In the test group, we observed significantly more EGFP expressing cells in the anterior olfactory nucleus (AON) than in the control group (Figures 2E,F and Supplementary Figures S4C,D). This is consistent with previous reports showing a TMT-induced activation of the AON (Saito et al., 2017). To examine the input specificity of TRACE, we quantified EGFP-labeled cells in selected regions, which send pronounced projections to the CoA, as characterized by our activity-independent AAV2-retro labeling (data not shown). However, we did not observe a difference between the control and the test groups (Supplementary Figure S3). This indicated that labeling is specific to the activated regions and merely projecting to a target region is not sufficient for EGFP labeling.

## Discussion and Outlook

Considering the complexity of the circuits underlying behaviors, unbiased approaches are particularly valuable in identifying the inputs which drive behavioral responses. Our results show that TRACE can identify active afferent inputs with virtually no need for *a priori* knowledge of their origins. Our approach, in principle, can be combined with genetically-encoded calcium indicators such as GCaMP to monitor the activity of input regions for different behavioral tasks. Also, TRACE can be used to deliver chemo- and optogenetic tools to the functionally relevant input neurons for further circuit manipulations. Since TRACE is a non-transsynaptic tracer, it is well suited for identifying neuromodulatory inputs that convey their signals through volume transmission rather than direct synaptic connections (Werner and Mitterauer, 2013). A potential concern in using a non-transsynaptic tracer as TRACE could be represented by the infection of axons passing through the injection site. However, there has not been such a report for AAV2-retro so far (Tervo et al., 2016).

In using TRACE, the same considerations must be taken as those for activity-dependent promoters and virus-mediated labeling. Viral tropism has been a recurring concern in investigating circuit mapping (Tervo et al., 2016; Sun et al., 2019). New strategies such as receptor complementation have been introduced to overcome the problem with tropism (Li et al., 2018). Alternatively, AAV2-retro can be complemented with a retrograde virus of different tropisms, such as canine adenovirus type-2. Similarly, activity-dependent promoters display region specificity (Guenthner et al., 2013; Denny et al., 2014), as TRACE was not efficient in labeling the olfactory bulb as a source for aversive input to the CoA despite its role in odor-driven innate aversive behavior (Root et al., 2014). Additionally, we and others have observed tamoxifen-independent labeling in selective regions such as layer 6 neurons within the neocortex and granule cells in the dentate gyrus in ArcCreERT2 transgenic mice (Guenthner et al., 2013). As such, all the experiments must be complemented with a control group in which mice are subjected to equal treatments except the test stimulus. However, with the rapid development in activity-dependent labeling systems, we are closer than ever to gain brain-wide access to neurons activated by a particular experience (Guenthner et al., 2013; Kawashima et al., 2013; Denny et al., 2014; Sørensen et al., 2016; DeNardo et al., 2019). We anticipate that TRACE, as it stands, will be generalizable and complementary to other circuit analysis methods for elucidating how neuronal activity in connected ensembles drives complex behaviors.

## Data Availability Statement

All datasets generated for this study are included in the article/Supplementary Material.

## Ethics Statement

The animal study was reviewed and approved by the Danish Animal Experiment Inspectorate.

## Author Contributions

The project was designed by SN and NK. The manuscript was written by SN, MC, and NK. Figures were designed and composed by NK, NM-J, and VK. For HFS and TMT testing, NK and NM-J performed the surgeries. For the foot-shock experiments, surgeries were done by NK. VK performed the TMT behavior. NK and MJ performed the HFS behavior. NK, MJ, and A-KV performed the foot-shock behavior. Imaging for HFS and foot-shock was done by NK and MJ. Imaging for TMT experiments was done by VK and NM-J. Cell counting was done by VK, MJ, and NK. Statistical analysis and graphs were done by NK and VK. SN and MC supervised the research. All authors discussed the results and contributed to the revision of the manuscript.

## Conflict of Interest

The authors declare that the research was conducted in the absence of any commercial or financial relationships that could be construed as a potential conflict of interest.

## Supplementary Material

FIGURE S1 Virus injection sites in mice used in this study. **(A)** The injection site for AAV2-retro-DIO-EGFP in LA, fiber placement above LA and injection site for AAV8-oChIEF-tdTomato in the TeA/Ect. **(B)** Injection site for AAV2-retro-DIO-EGFP in LHb. Injection site for FluoSpheres and AAV2-retro-DIO-mCherry in NAcc. **(C)** Injection site for AAV8-oChIEF-tdTomato in CoA (scale bar: 100 μm).

FIGURE S2 TMT exposure decreases the time spent in the dark chamber and increases the open field exploration. **(A)** Behavioral tracking using ANY-maze. The control group exposed to PBS showed no significant difference in time spent in the dark chamber during the last habituation phase vs. testing [*n* = 7; *p*-value = 0.2188 (Wilcoxon test)]. **(B)** Behavioral tracking using ANY-maze. The TMT exposed group showed a significant decrease in time spent in the dark chamber when exposed to TMT in comparison to the last habituation [*n* = 9; *p*-value < 0.0001 (paired *t*-test)]. Not shown: The percentage of time spent inside the box during habituation did not significantly differ between the PBS and TMT exposed groups [PBS group *n* = 7, TMT group *n* = 9; *p* = 0.1413 (Mann–Whitney test)]. n.s., not significant, *****p* < 0.0001.

FIGURE S3 Representative images of the CoA-projecting regions, which deemed insignificant by TRACE in mice exposed to TMT vs. PBS. No significant difference of labeling in the CoA-projecting neurons within: **(A)** anterior cingulate cortex area 32 [PBS *n* = 5, TMT *n* = 6; *p*-value = 0.4242 (Mann–Whitney test)], **(B)** substantia nigra reticulata [PBS *n* = 4, TMT *n* = 6; *p*-value = 0.2429 (Mann–Whitney test)], **(C)** medial orbital cortex [PBS *n* = 5, TMT *n* = 6; *p*-value = 0.5281 (Mann–Whitney test); scale bar: 100 μm]. n.s., not significant.

FIGURE S4 **(A)** Representative images of the LA, outlined with the white line, in mice receiving HFS or no stimulation. Green shows activity-mediated EGFP fluorescent expression. Blue shows DAPI stained nuclei (scale bar: 100 μm). **(B)** Representative images of the TeA/Ect in mice receiving HFS or no stimulation. Green shows activity-mediated EGFP fluorescent expression in the LA-projecting neurons within TeA/Ect. Blue shows DAPI stained nuclei (scale bar: 100 μm). **(C)** Magnified images of the CoA in mice exposed to PBS or TMT. Green shows activity-mediated EGFP fluorescent expression. Blue shows DAPI stained nuclei (scale bars: 100 μm). **(D)** Magnified images of the anterior olfactory nucleus (AON) in mice exposed to PBS or TMT. Green shows activity-mediated EGFP fluorescent expression in the CoA-projecting neurons within AON. Blue shows DAPI stained nuclei (scale bar: 100 μm).
