# Supplementary figures and images for: TRACE: An Unbiased Method to Permanently Tag Transiently Activated Inputs
Source: Front Cell Neurosci. 2020 May 12;14:114. doi: 10.3389/fncel.2020.00114 (PMC7243865; doi:10.3389/fncel.2020.00114)

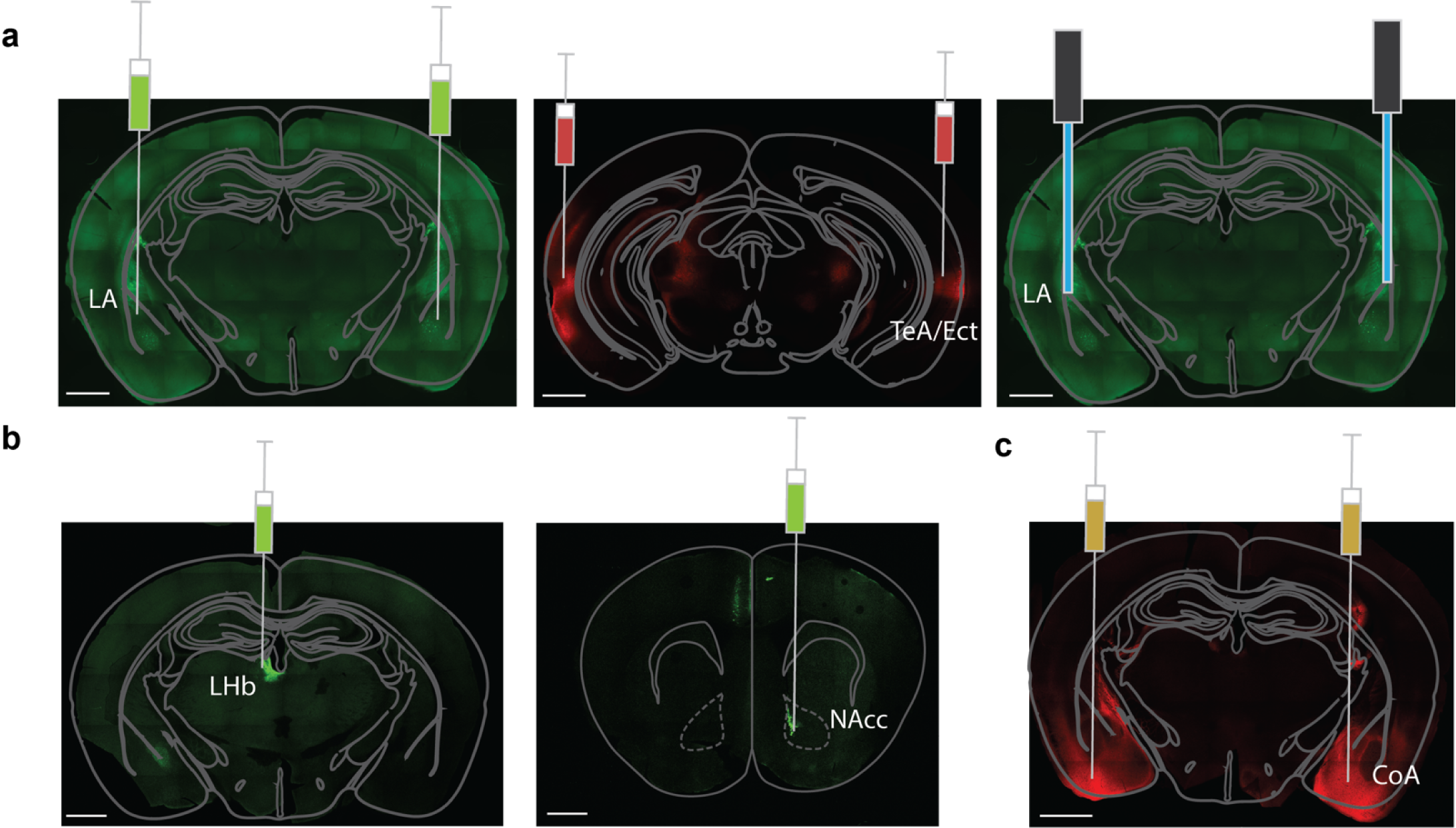

Supplement: FIGURE S1 — Virus injection sites in mice used in this study. (A) The injection site for AAV2-retro-DIO-EGFP in LA, fiber placement above LA and injection site for AAV8-oChIEF-tdTomato in the TeA/Ect. (B) Injection site for AAV2-retro-DIO-EGFP in LHb. Injection site for FluoSpheres and AAV2-retro-DIO-mCherry in NAcc. (C) Injection site for AAV8-oChIEF-tdTomato in CoA (scale bar: 100 μm). [file Image_1.TIF]

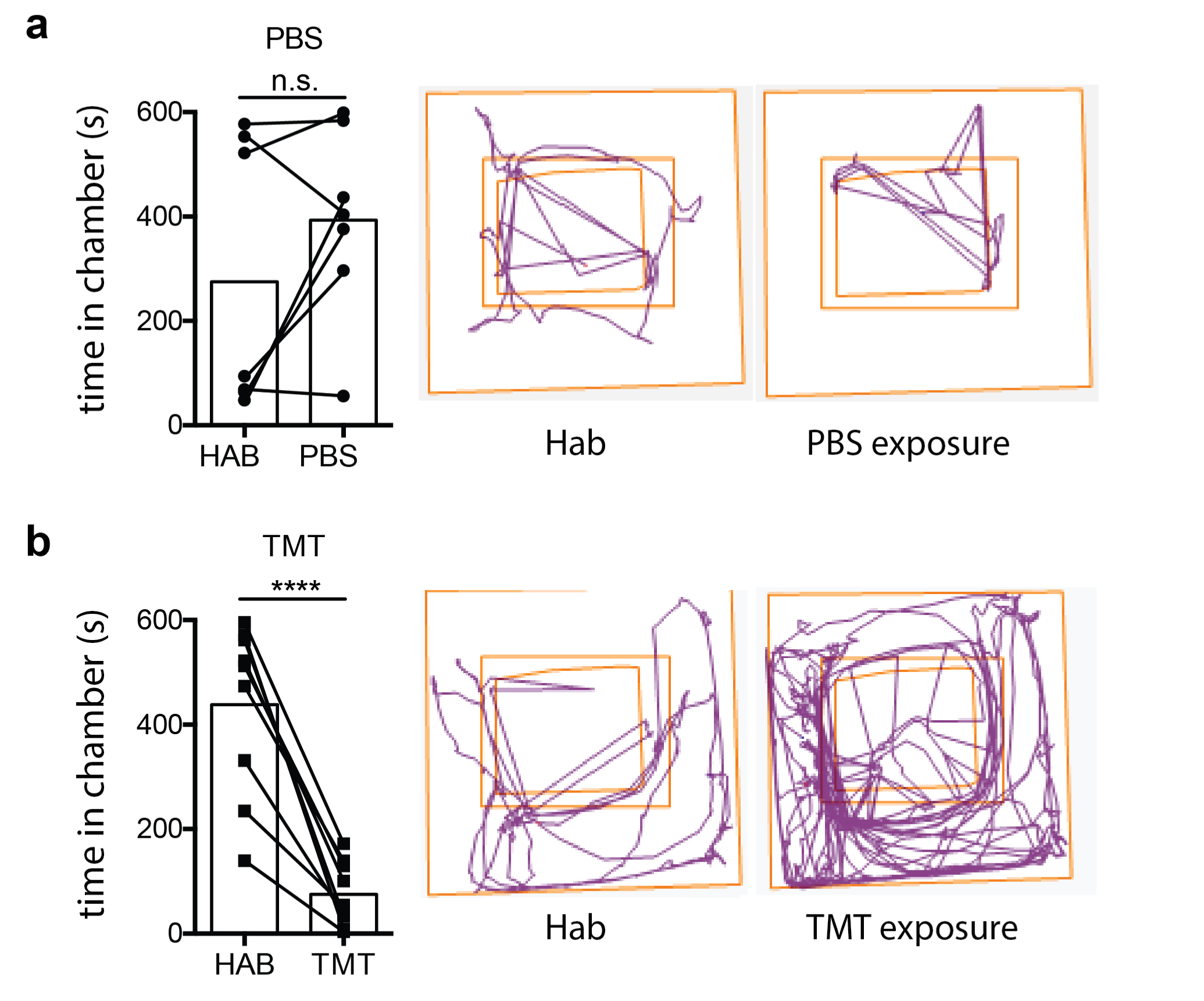

Supplement: FIGURE S2 — TMT exposure decreases the time spent in the dark chamber and increases the open field exploration. (A) Behavioral tracking using ANY-maze. The control group exposed to PBS showed no significant difference in time spent in the dark chamber during the last habituation phase vs. testing [n = 7; p-value = 0.2188 (Wilcoxon test)]. (B) Behavioral tracking using ANY-maze. The TMT exposed group showed a significant decrease in time spent in the dark chamber when exposed to TMT in comparison to the last habituation [n = 9; p-value < 0.0001 (paired t-test)]. Not shown: The percentage of time spent inside the box during habituation did not significantly differ between the PBS and TMT exposed groups [PBS group n = 7, TMT group n = 9; p = 0.1413 (Mann–Whitney test)]. n.s., not significant, ****p < 0.0001. [file Image_2.TIF]

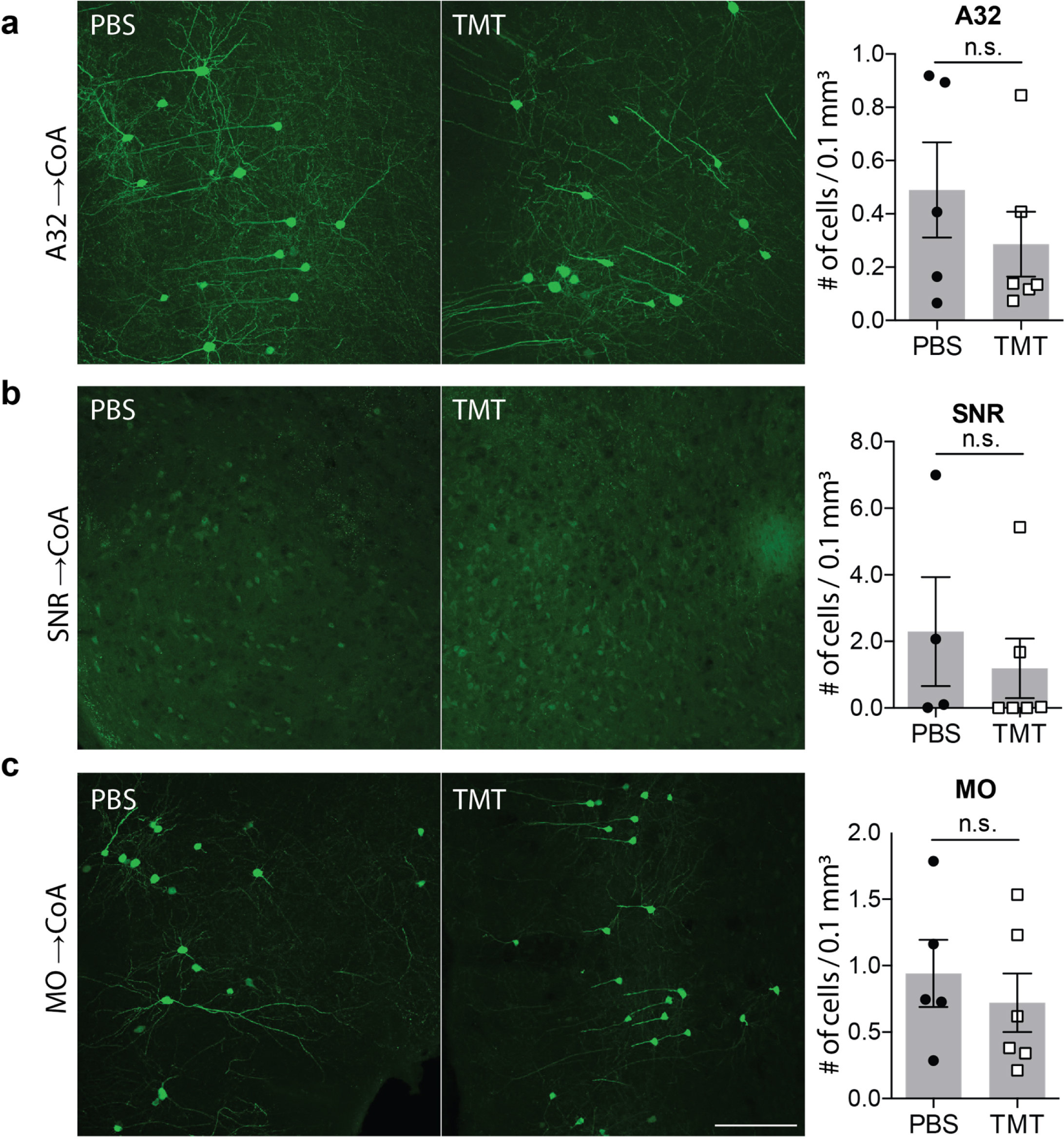

Supplement: FIGURE S3 — Representative images of the CoA-projecting regions, which deemed insignificant by TRACE in mice exposed to TMT vs. PBS. No significant difference of labeling in the CoA-projecting neurons within: (A) anterior cingulate cortex area 32 [PBS n = 5, TMT n = 6; p-value = 0.4242 (Mann–Whitney test)], (B) substantia nigra reticulata [PBS n = 4, TMT n = 6; p-value = 0.2429 (Mann–Whitney test)], (C) medial orbital cortex [PBS n = 5, TMT n = 6; p-value = 0.5281 (Mann–Whitney test); scale bar: 100 μm]. n.s., not significant. [file Image_3.TIF]

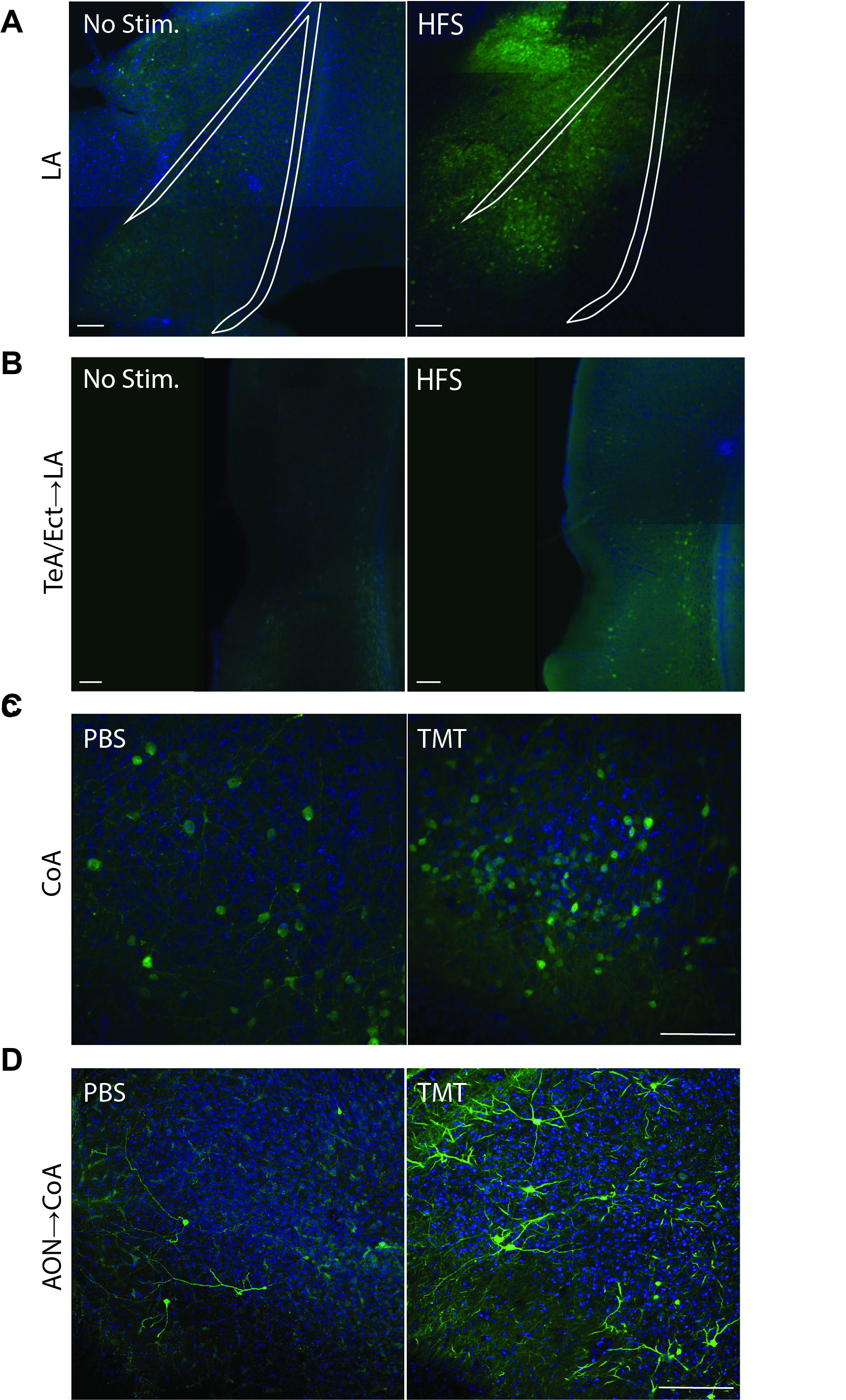

Supplement: FIGURE S4 — (A) Representative images of the LA, outlined with the white line, in mice receiving HFS or no stimulation. Green shows activity-mediated EGFP fluorescent expression. Blue shows DAPI stained nuclei (scale bar: 100 μm). (B) Representative images of the TeA/Ect in mice receiving HFS or no stimulation. Green shows activity-mediated EGFP fluorescent expression in the LA-projecting neurons within TeA/Ect. Blue shows DAPI stained nuclei (scale bar: 100 μm). (C) Magnified images of the CoA in mice exposed to PBS or TMT. Green shows activity-mediated EGFP fluorescent expression. Blue shows DAPI stained nuclei (scale bars: 100 μm). (D) Magnified images of the anterior olfactory nucleus (AON) in mice exposed to PBS or TMT. Green shows activity-mediated EGFP fluorescent expression in the CoA-projecting neurons within AON. Blue shows DAPI stained nuclei (scale bar: 100 μm). [file Image_4.tif]
